# Micro-regional planning: evidence-based community buy-in for health development in five of Mexico’s poorest rural districts

**DOI:** 10.1186/1472-6963-11-S2-S2

**Published:** 2011-12-21

**Authors:** Ascencio Villegas Arrizón, Neil Andersson, Robert J Ledogar

**Affiliations:** 1 Centro de Investigación de Enfermedades Tropicales, Universidad Autónoma de Guerrero, Calle Pino, El Roble, Acapulco, Mexico; 2 CIETinternational, 511 Avenue of the Americas #132, New York, NY 10011, USA

## Abstract

**Background:**

Community participation was a core tenet of Primary Health Care as articulated in the 1970s. How this could be generated and maintained was less clear. This historical article describes development of protocols for evidence-based community mobilisation in five local administrative units (*municipios*) in the Mexican state of Guerrero between 1992 and 1995.

**Methods:**

A sample of five to eight sentinel sites represented each of the most impoverished municipalities of the poorest five of the state's seven regions. A 1992 baseline survey of diarrhoea and its actionable determinants provided the substrate for discussion with local planners and communities. Municipal planners used different strategies to promote participation. In one municipality, new health committees took control of water quality. In another, municipal authorities hired health promoters; a song promoted oral rehydration, and house-to-house interpersonal discussions promoted chlorination. In the poorest and most mountainous municipality, *radio casera* (home-made radio) soap operas used local "stars". In the largest and most disparate municipality, a child-to-family scheme relied on primary and secondary school teachers. The research team assessed outcomes at intervals and used the results to reinforce local planning and action.

**Results:**

Diarrhoea rates declined in all five municipalities, and there were several positive intermediate outcomes from the communication strategies – changing knowledge, household practices and uptake of services. There was a strong link between specific contents of the communication package and the changing knowledge or practices.

**Conclusions:**

Apart from these evidence-based interventions, other factors probably contributed to the decline of childhood diarrhoea. But, by monitoring implementation of planning decisions and the impact this has at community level, micro-regional planning can stimulate and reinforce actions likely to improve the health of communities. The process empowered municipalities to get access to more resources from the state government and international agencies.

## Background

A micro-region is a distinct territorial unit with clearly marked boundaries below the regional level, but above the village level [1]. In Latin America the most natural micro-region is the municipality, the most peripheral local administrative and political unit, similar to a district in other countries. Micro-regional planning is generally a sub-discipline of regional planning [2], which in turn is largely concerned with land use [3]. We use the term in an analogous sense, concerned with socio-economic development, but with a particular focus on population health. We consider that planning is best if evidence-based and the evidence we use most directly comes from epidemiological research.

From its inauguration in 1986, the Centre for Tropical Disease Research (CIET) of the *Universidad Autónoma de Guerrero* has developed methods for evidence-based planning in Mexico's Guerrero State. One of its earliest applications involved many of the poorest municipalities in Guerrero between 1992 and 1995. CIET entered into agreement with local governments in the five poorest municipalities in the State to develop an evidence-based local planning system. This report describes the experience that we called micro-regional planning, and its results. Although the five municipalities are quite different from one another culturally and topographically, they had in common that each was the most underdeveloped of its region (see Table 1 and Additional file 1). They are all geographically, culturally, economically and politically remote from the main centres of Guerrero State, one of the three poorest states in Mexico. Health conditions in the five municipalities at the time of this project were comparable to the worst in other developing countries. Infant mortality was nearly 100 per 1000 live births; more than one half of adult women were illiterate; and less than one household in ten had ready access to drinking water. Only 27 of the 700 distinct communities had a health centre [4].

**Table 1:** General characteristics of the municipalities in 1992

Municipality	Population and ethnicity	Main productive activities	Migratory destination	Health centres
**Zirándaro** (400 communities)	21,300 Mestizo	Farming, Cattle-raising Fisheries	USA	7
**Copalillo** (12 communities)	11,122 Nahual	Farming, Handicrafts	Nayarit, Sinaloa Morelos	5
**Alcozauca** (25 communities)	15,089 Mixteco	Farming Handicrafts	Sinaloa USA	5
**Coahuayutla** (167 communities)	13,461 Mestizo	Farming, Livestock Fisheries	USA	5
**Xochistlahuaca** (103 communities)	16,226 Amuzgo	Farming, Handicrafts	Acapulco	5

Micro-regional planning sought to increase the community stake-holding in planning through informed participation in fact-finding on immediate health problems, focussed on specific and affordable actions. Whatever the longer term achievements, we hoped to precipitate action likely to have a short-term impact, and by measuring that impact, to build confidence of municipal and household decision makers in basing decisions on hard local evidence. A secondary aim was to define and to test communication techniques to promote participation that solves problems identified. Another secondary aim was to train local people in evidence-based planning, so that the process could continue after completion of the externally financed project.

## Methods

Translated into practice, micro-regional planning means a set of local research activities repeated at intervals to support evidence based planning decisions. In the CIET approach these progressively include local authorities and key decision-makers, while sustaining scientific rigour. Each cycle began with identification of actionable problems, like diarrhoea, water supplies, hygienic practices in the household and oral rehydration. Analysis of existing data on this set of problems and identification of information gaps formed the basis of the cycle design. Design was a collaborative and participatory process, involving local civic leaders, health workers and often school teachers. The researchers accompanied the process throughout, facilitating steps (like design groups and pilot testing) that did not have local protocols (as do community meetings).

We have described sampling in detail elsewhere [5,6]. In this project, 46 sentinel sites represented around 700 named settlements (10 in Zirándaro, 11 Copalillo, 7 Alcozauca, 8 Coahuayutla and 8 Xochistlauaca). Exclusion of around 400 settlements with fewer than 30 residents meant the sentinel sites were bigger than the average settlement size; the 20,000 people in the sample represented around 20% of the total population of the regions.

Field work involved several linked research methods in the same panel of communities: (i) household questionnaires, perhaps enquiring about children or women; (ii) an institutional review of the nearest school or health centre – often named by the household respondents; (iii) rapid assessment procedures to generate qualitative evidence on the site; and (iv) focus groups of community members (separate for men and women) and sometimes service workers, to interpret results, to develop communication strategies and to formulate workable local actions. A separate publication details these steps, who does them, and how [7]. In this project, researchers trained local interviewers, typically young people from the nearest college (*preparatoria*), who could work in the local language but who could also communicate in Spanish.

In each data-collection cycle, we compiled evidence on one health problem and its solutions in a panel of communities (clusters) representing each municipality. Community members in focus groups and community meetings selected components of morbidity and mortality on which low-cost interventions might have an impact.

The principal theme of the first cycle was diarrhoea. In a 1989 survey of 3,603 children under three years old in 43 sentinel sites throughout the state of Guerrero, CIET found that some 34% of children under three years old had suffered from diarrhoea in the two weeks before the survey. Questions about risk factors revealed that 50% of the families kept pigs in the home, 58% had no lavatory facilities and 49% had no running water in the home. A child who drank unpurified water had a three times greater risk of getting diarrhoea than a child who drank purified water. Half of the mothers gave their sick child less food than usual or no food at all during the episode of diarrhoea and less than half of them gave their sick child extra liquids. Those children whose mothers gave them less liquids than usual during an episode of diarrhoea had a 50% greater risk of the episode being prolonged [8].

The analysis started by establishing the frequency of occurrences in the community. In the first cycle, one child in every three had recent diarrhoea and two out of three households did not use latrines. Subsequently, the analysis identified associations susceptible to change, using stratification to define and to take into account epidemiological confounders and effect modifiers [9,10]. A central theme in analysis and in subsequent discussions was the reported cost to the community and to the services of each intervention, and the reported cost of failure to intervene [11]. We measured household costs of health care in several ways. We estimated household costs for preventive activities, like latrines or boiling water. For each case of diarrhoea, we asked the mother how much they spent in cash and how much time they spent caring for the sick child. We ascertained costs of often used drugs from local merchants. We summarised reported costs of diseases in terms of working days lost as a result of illness or looking after a sick person.

The researchers first carried out the mechanical aspects of analysis (tabulation of data, analysis of risk factors, and computation of costs). Then, in preparation for socialisation of the results, they discussed these results in focus groups and small community meetings, with community members playing the key role in interpretation.

The practical challenge of the project was to share and to discuss the results in other communities in the municipality beyond the sample communities, attempting to provoke decisions and action based on measured need and potential impact of interventions. The idea was not to tell people what is good for them, but to show them how the analysis played out, and to let them draw their own conclusions. The field team packaged findings for feedback, often without leaving the community, in a series of steps that gradually became standardised: computational analysis; consultative interpretation, often through focus groups and small meetings; framing emerging themes to share; development and testing of numeric and narrative formats; and then dissemination.

Micro-regional planners used several channels to socialise evidence. Most favoured were community meetings and summary reports (a single page of preliminary results, with 50 to 60 words in very large type). After the first cycle of data gathering, community consultation played a key role in deciding which communication strategies were most suited to each municipality. Researchers paid increasing attention to the use of numbers and the ways communities can understand numbers, risks and potential gains.

In Xochistlahuaca, the best communication mechanism was through community meetings, at which street theatre (known locally as *sociodrama*) played by voluntary promoters in all communities (sentinel and otherwise) in each cycle. In Zirándaro, the team used street theatre to convey results to the communities, on some occasions through groups of children using home-made puppets. In Copalillo, a local municipal official coördinated feedback and discussion of results about a water cost study, including preparation of a booklet.

In the more remote mountain areas, people preferred radio messages and hand drawn cartoon "comics". In Alcozauca in La Montaña region, at different times of the day, the local community loudspeakers transmitted *Radio Casera* (literally, "Home-made Radio"); among its programmes were tape-recorded soap operas featuring voices of people from the local community, presenting the results or the argument being proposed, overlaid with humour and pathos.

In the communication activities, the researchers accompanied the process and played a facilitating role with community members taking the lead in generating ideas and implementing them. As the researchers had by this stage lived in the communities for some time, they joined in the activities along with community members, instead of observing from the margin.

Results of the first survey cycle not only served as a baseline for final evaluation of the project but also provided evidence for feedback to engage communities. Diarrhoea was common in all municipalities, but the risk factors for diarrhoea differed between municipalities. . There was a robust association between diarrhoea and water treatment in some places, while in others there was an association between diarrhoea and presence of latrines. For example, based on the risk difference (26 percent) for diarrhoea in households with and without latrines, the community consultation in Alcozauca chose latrine installation as the intervention likely to maximise impact.

The results of the first cycle and the opinions of community leaders and members who took part in the discussions influenced the choice of focus for the second cycle (See Results section).

### Ethical considerations

The CIET ethics committee considered the ethical issues and approved the study. As only 27 of the 700 localities in the five municipalities had a health centre, we decided from the beginning to offer free medical consultation at the time of the survey. Most of the health units had no physician and people received medical care from a health auxiliary, generally a person from the community acting as a nurse. Though for poor people in a remote community two days of access to medical consultation per year is unlikely to change morbidity and mortality patterns, it does represent an intervention, in the sense that other similar communities do not have even this minimal service. We considered that, to comply with ethical imperatives, the potential bias introduced by these medical consultations was acceptably small.

## Results

### Improvement of knowledge and practices related to diarrhoea

In the five municipalities, field teams surveyed some 3,000 households each cycle, around 20 percent of the entire population of the municipalities (Table 2).

**Table 2:** Number of people and households surveyed in 1992, 1993 and 1995

		1992	1993	1995
Zirándaro	Households	722	632	700
	Individuals	4078	3239	3800
	Groups		8	10

Alcozauca	Households	730	670	720
	Individuals	5052	5094	5401
	Groups		7	9

Xochistlahuaca	Households	784	399	595
	Individuals	4382	2263	3349
	Groups		9	8

Copalillo	Households	976	732	867
	Individuals	6380	4805	6154
	Groups		12	15

Coahuayutla	Households	539	503	512
	Individuals	2874	2837	2711
	Groups		8	8

TOTALS	Households	3751	2936	3394
	Individuals	22715	18238	21515
	Groups		44	50

Tables 3, 4 and 5 show changes in health knowledge and practices. In most communities, water treatment, by boiling or chlorination, increased over the period. Knowledge of how to prepare home-made oral rehydration solution for cases of diarrhoea increased in four of the five municipalities in the first year but later declined in all but one of them (Table 6). Over the same period, there was a fairly steady improvement in the proportions of children whose parents gave them the same amount or more liquids than usual when they had diarrhoea (Table 4). This improvement occurred even in municipalities where knowledge of ORS preparation fell between 1993 to 1995 (Table 6).

**Table 3:** Percentage of households who either boiled or chlorinated their drinking water

	Percentage (fraction) that boiled or chlorinated water			p-value (2df)
	1992	1993	1995	
Zirándaro	16 (116/722)	27 (171/632)	37 (259/700)	<0.05
Alcozauca	12 (88/730)	41 (275/670)	27 (194/729)	<0.05
Xochistlahuaca	11 (86/784)	41 (163/399)	55 (327/595)	<0.05
Copalillo	43 (420/976)	44 (322/732)	60 (520/867)	<0.05
Coahuayutla	39 (210/539)	52 (262/503)	49 (251/512)	<0.05

**Table 4:** Children with diarrhoea who were given the same or greater quantity of liquids

	Number of children with diarrhoea (percentage given the same or more fluids than usual)		
	1992	1993	1995
Zirándaro	128 (23)	60 (25)	23 (40)
Alcozauca	347 (53)	271 (66)	214 (60)
Xochistlahuaca	213 (73)	73 (83)	74 (70)
Copalillo	195 (35)	76 (49)	224 (90)
Coahuayutla	123 (70)	117 (80)	94 (93)

**Table 5:** Proportion of households who made use of latrines, 1992-1995

	Percentage (fraction) that used latrines			p-value (2df)
	1992	1993	1995	
Zirándaro	5 (36/722)	5 (32/632)	7 (49/700)	0.18
Alcozauca	4 (29/730)	13 (87/670)	16 (115/720)	<0.05
Xochistlahuaca	8 (63/784)	32 (128/399)	24 (143/595)	<0.05
Copalillo	16 (156/976)	16 (117/732)	15 (130/867)	0.81
Coahuayutla	4 (22/539)	5 (25/503)	16 (82/512)	<0.05

**Table 6:** Proportion of households that could correctly describe preparation of oral rehydration salts

	Percentage (fraction) that could describe preparation of ORS			p-value (2df)
	1992	1993	1995	
Zirándaro	33 (238/722)	42 (265/632)	34 (238/700)	Ns
Alcozauca	12 (88/730)	17 (114/670)	15 (108/720)	Ns
Xochistlahuaca	10 (78/784)	28 (112/399)	30 (178/595)	<0.05
Copalillo	17 (166/976)	14 (102/732)	9 (78/867)	<0.05
Coahuayutla	20 (108/539)	45 (226/503)	9 (46/512)	Ns

From a very low starting point, latrine use improved fairly consistently in three of the five municipalities from 1992 to 1995. In Copalillo it remained unchanged and in Xochistlahuaca, after remarkable improvement from 1992 to 1993, it declined in 1995.

**Figure 1:**
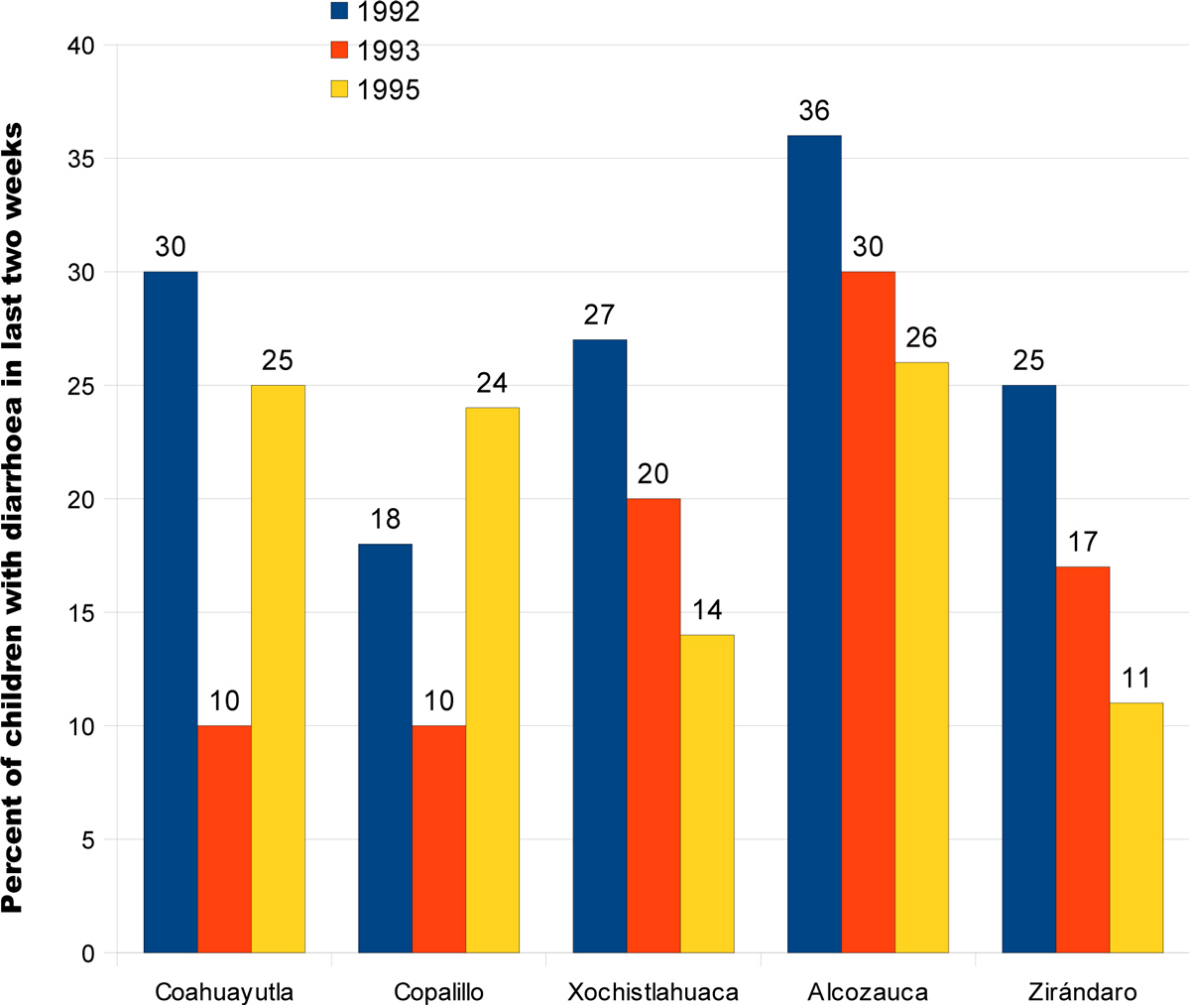
**Childhood diarrhoea rates (% children who suffered diarrhoea in the last two weeks) in five municipalities in 1992, 1993 and 1995**. Blue = 1992, Orange = 1993, Yellow = 1995

Diarrhoea prevalence declined sharply between 1992 and 1993 in all municipalities (Figure 1). This decline continued into 1995 in three of the five municipalities but the trend reversed in the other two and in the case of Copalillo, the 1995 prevalence was higher than in 1992.

We found a range of costs for the average case of childhood diarrhoea across the municipalities, from M$12 in Alcozauca (one day at the official minimum wage), to M$33 in Copalillo (3 days). Municipal planners incorporated information on costs into discussions with the communities about what to do to decrease diarrhoea incidence.

### Capacity building and the community voice

CIET's epidemiology residents, one in each municipality, supported the capacity building effort. Three of the five municipalities supported in-service training of a "micro-regional planner" in the municipal administration. This person received training in research planning, questionnaire design, survey execution, data analysis, communication techniques and resource management. The other two municipalities incorporated community health promoters from the project in their planning process. The project trained a total of 96 health promoters. In Xochistlahuaca the municipal authorities opted to pay their health promoters for their continued services.

Lack of doctors in health centres was a common complaint from communities. During the second year of the project, the government assigned doctors to four of the five previously abandoned health centres in two municipalities. In both Xochistlahuaca and Alcozauca the communities formally denounced the failure of the health alderman to chlorinate the water.

Several municipalities identified other local problems they wished to study. Three of the five municipalities decided to study scorpion sting, for them an important and unresolved health problem not addressed adequately by traditional or Western health care systems. In Copalillo, the municipal authorities used results of the scorpion sting study to argue for introduction of electric power in three communities. The study had shown a higher incidence of scorpion sting during the night in communities without electric power.

Also in Copalillo, after the population and the authorities reflected on the cost of supplying households with piped water, a secondary study of costs of collecting water was undertaken, confirming that it would be cheaper to install a water outlet to each household than to continue with each household carrying water from the sources. These data led to a municipal agreement with the state government to build a pipe network.

In Alcozauca municipality, a programme of building latrines based on the study results gave rise to another development action: three communities repaired their access road to transport materials for the programme.

Many communities formed water chlorination monitoring committees, noting the increased risk for everyone from a contaminated well. In Xochistlahuaca, where the study identified an increased diarrhoea risk from allowing pigs to wander freely through the community, the population agreed for the first time to tether these free-range pigs (free-range pigs are the garbage and sewage disposal "system" of these areas).

Dialogue on study results in Alcozauca generated a proposal for a latrine-building project, later presented successfully to UNDP for funding. The municipal authorities negotiated the latrine micro-project with the *Instituto Nacional Indigenista* (INI), which channelled UNDP funds to buy cement while each community provided stone, sand, gravel and man-power. The CIET epidemiology resident in charge of the project, with the municipal planner (the municipal police officer, retrained as a planner in the course of the project), built one demonstration latrine in each of 16 communities and supervised construction of others. Radio messages with information about maintenance supplemented handmade booklets and posters explaining the construction. In less than four months, 16 of the municipality's 20 communities installed nearly 1,000 latrines.

## Discussion

The project undoubtedly increased the community stake-holding in planning through informed participation in fact-finding and generation of specific and affordable actions. Several of the interventions had a measurable short-term impact and, by measuring that impact, the project started to build confidence of municipal and household decision makers.

The project developed communication protocols and techniques that drew directly on the epidemiological analysis. Communication strategies and intensity varied between municipalities. The project also exposed many people to aspects of evidence-based planning, including ordinary community members, who provided evidence, discussed that evidence, and proposed and implemented corrective actions.

### Study limitations

Methodological concerns in participatory evidence-based planning can be different from those in academic research [12,13]. To start with, involvement and commitment of stakeholders is necessary in evidence-based planning, where academic research might try to limit this influence. Evidence-based planning efforts often do not have a control population against which to measure the impact of interventions. More recent approaches include control populations, as in ongoing CIET work in Nigeria [14]. The real life setting with other actors active in the communities -- government, non-governmental organisations, private health providers, churches – means that few if any of the positive outcomes over the period of the project could be attributed solely to the evidence-based planning project and its interventions.

We are unable to separate out impacts of the different activities – like the impact of the cartoons versus the impact of the *radio casera.* There was considerable overlap in the same sites and it is quite possible that cartoons tipped the balance in one place while *radio casera* did so in another. Presentation of the evidence keyed off a number of communication ideas in the communities; supporting communities to bring their own communication ideas to life seems to have had a broadly positive effect.

We are cautious not to over-interpret that positive effect, given the constraints of our design. Sites were not randomly allocated to intervention, there were no control sites, and life continued in its characteristically uneven way, with cholera epidemics in some places, making changes over time hard to interpret. We focus rather on the increased stake-holding of communities in evidence-based planning, the confidence in evidence among municipal decision makers, and the local capacities built.

There are problems of using epidemiology in remote and resource constrained settings. In successive research cycles, the number of households contacted during the survey was smaller in some municipalities. One result of local participation in design was that the questionnaire in the second cycle was longer than in the first, resulting in a lower number of households contacted. Another reason for the reduced number of households contacted was migration of families due to the economic crisis; in two of the mountain communities in the sample, nearly the entire village population left the area.

Migration in these worst-off municipalities presented a potential measurement problem, distorting apparently positive results; among other effects, migration of the worst off may cause an under-estimation of mortality and morbidity rates. To the extent that this migration is constant and documented household by household, however, evidence generated by the successive cycles could still be interpreted correctly. The interview records permitted identification of every household; it was possible, therefore, to consider the characteristics of households that migrated and those that did not. The follow-up questionnaire, covering 34,000 person years, also permitted detailed analysis of survival and disability associated with differential access to specific health care interventions.

In the early stages, the project generated and disseminated too much evidence. The population lacked capacity to assimilate and to act upon all of it. The different problems in different municipalities during the early stages of the work distracted from the anticipated multiplication of knowledge through exchange between municipalities. We adjusted the work plan in the second year because of the over-abundance of evidence produced by the first two cycles, before local action could resolve the problems identified. Focus group discussions in each site helped people to customise the evidence format from the first two cycles, introducing local concepts and numerical formats easily understood in the communities. By the end of the second year, a steadier rhythm permitted fuller discussion and more effective action.

Diarrhoea occurrence declined in three of the five municipalities but we would not claim this decline was only because of evidence-based planning and its attendant community engagement. Resurgence of diarrhoea in Copalillo might be explained by a cholera outbreak that coincided with the 1995 follow-up survey.

The contrast between diminishing knowledge of preparation of oral rehydration salts (ORS) and the increasing use of liquids during episodes of diarrhoea during the period from 1993 to 1995 (Table 4) is partly explained by a disagreement over strategy between municipal planners and the State Health Secretariat. The municipal planners had previously promoted ORS but, because the pre-packaged salts were not always available in sufficient quantities, especially in the more remote communities, they also taught households to prepare their own rehydration solutions (*suero casero*). The Secretariat opposed *suero casero* and promoted the use of ORS exclusively. A compromise was finally reached whereby the project would promote both remedies and the Secretariat would supply greater quantities of ORS to the more remote communities.

Experience with latrines illustrates that evidence can stimulate action but also that changing some behaviour patterns takes sustained effort. Latrine use over the life of the project was disappointingly low. One explanation given for this is that people in these communities usually inspect their excreta for signs of their health status and latrines make this impossible. The development literature abounds with examples of failed latrine projects [15].

### The planning dynamic and community voice

The project shows that micro-regional planning can generate actions likely to improve the health of communities. It is possible to engage local administrations in community-based research and to socialise the results beyond the sentinel sites to precipitate problem-specific decisions. Being able to monitor implementation of their decisions, and the impact this has at community level, reinforces the entire process.

A perhaps predictable but still useful result was the extent to which specific, locally-relevant evidence did actually change knowledge, attitudes and practices. A more surprising and encouraging result was the way these changes empowered communities. The state government assigned physicians to previously abandoned health centres and the communities denounced of the failure of heath aldermen to chlorinate water – these are examples of increased community voice.

When translated through focus groups into local terminology and numerical formats, communities easily assimilated concepts of the relationship between risk factors and disease. For example, many people came to see untreated water and having pigs run around loose as risk factors for diarrhoea. Many communities saw the need for water treatment. The concept of relative risk proved useful at household level, helping parents to understand how much they could decrease the risk of diarrhoea for their child by treating water in the home. For planners needing to choose based on greatest potential benefit for the population, risk difference was more useful. In Alcozauca, the research team explained to the municipal planners that a programme of building latrines could potentially decrease the childhood diarrhoea rate by 16 percent.

### Consciousness about costs

The project led to increased consciousness in communities about cost of diseases and costs of prevention activities. We quantified household costs of illness in each municipality, and discussed these costs with the communities. The cost data provided another stimulus for action to prevent common illnesses like diarrhoea. Households and planners could compare what they would spend on prevention with what they spent on treatment. Children questioned their parents about family expenditures on water chlorination, and about how many people and how much time it took to supply the home with water.

Cost-consciousness about water supply led some communities to the conclusion that it would be cheaper to install a water outlet to each household than for households to continue carrying water from the sources in the main town. Once communities knew the results about costs, it became difficult to avoid discussing costs, including those beyond health – like women's time.

### Health promoters and local health care capacity

One aim of the micro-regional planning project was to clarify practical issues in decentralising health research and in sustaining decision-making based on the results of this research at the municipal level.

One of the first demands of the communities was medical care. They resented having no access to qualified health workers. . For ethical reasons, the project provided some basic medical care activities in the communities at the time of the surveys. Although this was not a project goal, providing this medical care drew attention to the deficiencies of the existing medical care services.

At the beginning of the first cycle, only two municipalities had any doctors assigned to their health centres. During and after the project, communities continued to demand doctors for their health centres. By 2008, Xochistlahuaca alone had five doctors assigned to its health centres.

As a partial response to community demands for medical care, the project developed a programme to train community health promoters in aspects of curative and preventive care. Some 48 communities appointed a total of 96 promoters. Most of them already had a health role in the community, such as traditional healers, midwives, health auxiliaries. The project trained some promoters in water chlorination, treatment of scorpion stings, and construction and maintenance of latrines. In Xochistlahuaca, five promoters "graduated" into the role of micro-regional planners. Their numbers increased over the years and in 1996 they formed the Indigenous Amuzgo Health Promoters Network.

The role of community health promoters in developing countries has grown since the Alma-Ata conference on primary health care in 1978. This trend has spread to developed countries more recently [16]. The promoters trained through this project acquired a particular set of skills, tailored towards sharing evidence with communities and responding to the specific needs identified by the evidence.

Training of the promoters and local medical staff focussed on the topic of the research cycles. Promoters also participated in data collection, preliminary analysis, and dissemination of results. Among indigenous communities, promoters were the linguistic connection in meetings and focus group discussions. They received training in counselling people in the community.

As a secondary effect, the work drew attention to deficiencies of health care at the periphery. In these remote communities, giving a patient a medical prescription means nothing as there are few places to buy the drugs and people seldom have resources to do so anyway. A partial solution arising from this project were the "popular pharmacies", a Bamako initiative-style [17] solution involving rotating funds for purchasing a limited range of essential drugs.

### Heterogeneity

Despite exactly the same basic intervention and a CIET epidemiology resident in each municipality, the response to the results varied greatly, with different communication channels and formats. Some communities absorbed the implications with almost disconcerting speed, participated actively in the search for solutions, and implemented remedial actions with their own resources or sought external resources to do the job. Others were much slower to act. This could not easily be explained by ethnic differences.

The political situation could have contributed to the heterogeneity in response between municipalities. Since the project covered municipalities run by the then ruling party (PRI), the opposition (PRD) and a smaller left-wing party (PRT), there were always some communities out of favour with the political leadership of the local administration. Outright rejection of all local government initiatives, particularly in predominantly indigenous municipalities, meant underachievement by the micro-regional planning initiative in these municipalities. In one municipality with marked political polarization, Coahuayutla, this phenomenon did not apply and people saw micro-regional planning as a non-party activity. Political tensions were an incomplete explanation of the differences in impact.

The armed insurgency in nearby Chiapas, which began in 1994 after decades of severely constrained resources, also influenced the heterogeneity between municipalities. The federal government channelled massive economic resources to indigenous populations, including three of the municipalities in this project. For example, the Xochistlahuaca administration was suddenly willing and able to support each health promoter trained in this project with a monthly stipend. Copalillo municipality supported several new water and electricity initiatives.

There was heterogeneity of responses even within the same municipality. For example, while many communities formed water chlorination monitoring committees, noting the shared risk from a contaminated well, others preferred chlorinating water in their own homes.

## Conclusions: micro-regional planning in other settings

Key to assessing the impact of micro-regional planning is the *Hawthorne effect* (where the "subjects" of the study change as they engage with the study)*.* The concern here is that the study process itself makes the study or sentinel communities different from non-sentinel communities, which receive only the information that comes out of the consultation or dialogue in the study communities.

In theory, the interactive data-gathering itself and the medical consultations provided by the project could produce a difference between sample and non-sample communities, leading to overestimation of the impact of the micro-regional planning intervention. The changes in the study communities could be partly due to the process of measurement as well as to the intervention being measured. In practice, two days of free consultation per year probably did little to decrease the burden of disease and disability in these impoverished communities. Socializing the survey findings and dialogue about these findings both in the study sites and in non-study sites by means of the communication strategy should have reduced any difference between study and non-study sites due to the study process itself.

This is the rub of micro-regional planning. It attempts to draw the public and politicians into a common research process, a dialogue on priorities and results, changing their perspectives by doing so. Insofar as this happens, micro-regional planning is a success. Spreading the dialogue around evidence beyond the sample clusters to all other households decreases the difference between the sample and the rest of the municipality.

CIET is an active health player in Guerrero State, a post-graduate, academic institution dedicated to community-based research. This influence is not incidental nor can it be "controlled", as one might control a confounding factor in a laboratory experiment. CIET epidemiology residents might have an unusual dedication to community-level development, a characteristic that cannot be presumed of all civil servants who might roll out similar initiatives in other settings. These factors affect the feasibility and costs of micro-regional planning, the techniques developed for generating dialogue around the results, and the lessons from communication of evidence to mobilise communities beyond those which participated in the actual measurement.

The aim of the micro-regional planning project, its reason for existing, was to consider whether evidence about impact, coverage and cost, when communicated adequately at community level, can work as a catalyst to improve health [18,19]. In this project, it was almost certainly the researchers' presence, household interviews, community discussions, and assorted communication tools that together produced the changes, rather than any specific wording about risk in a pamphlet. Many intervention studies try to avoid the Hawthorne effect; micro-regional planning succeeds by creating this effect. Dialogue around the results changed levels of knowledge, attitudes and opinions as a result of the micro-regional planning process, possibly before any health impacts resulting from specific interventions.

## Competing interests

All authors declare they have no competing interests.

## Authors' contributions

AVA led the field research; NA designed and directed the research; RJL did background research and wrote this article based on drafts by AV and NA.

## Supplementary Material

Additional File 1 Description of the municipalities in 1992 Click here for file
