# Supplementary material for: Micro-regional planning: evidence-based community buy-in for health development in five of Mexico’s poorest rural districts
Source: BMC Health Serv Res. 2011 Dec 21;11(Suppl 2):S2. doi: 10.1186/1472-6963-11-S2-S2 (PMC3332561; doi:10.1186/1472-6963-11-S2-S2)
Supplement: Additional File 1 — Description of the municipalities in 1992 [file 1472-6963-11-S2-S2-S1.pdf]

Additional File 1

Description of the municipalities in 1992

Zirándaro: Located in the Tierra Caliente region, its tough terrain is crossed by the southern part of the Sierra Madre mountain range. It includes 400 communities, with a dispersed population of 21,300 (1990 census). Farming and livestock form the basis of the economy; some irrigated farm lands yield corn and sesame seed. Some communities fish (in rivers) for their own consumption. A large part of the young population migrates seasonally to the United States seeking employment. Only seven of the 400 communities have a health centre: physicians staff five of these, nurses staff the other two. In the three largest towns, there are private physicians.

Copalillo: With its predominantly Nahuatl population, the municipality is made up of 10 communities 20 settlements and a total population of 12,000. Most live near rivers, their main activity being farming for their own consumption. Some weave hammocks, sold usually through intermediaries. There are five health centres, three without a physician and two private physicians in the municipal capital.

Alcozauca: Located in the Mountain Region, in the highest area of the southern Sierra Madre, 15,089 indigenous people of Mixteco ancestry live in 20 communities that are outstanding for their isolation and underdevelopment. Dry land corn farming for self-consumption and weaving straw hats provide the means for survival. Few employment opportunities exist and seasonal migration is common. After the harvest, up to 50 percent of families migrate to Sinaloa, where they are hired as agricultural labour, returning four months later to prepare for the new farming cycle. Many leave for the United States in search of work. There are five health centres, one staffed by a physician. There is another physician in private practice and a pharmacy. Almost all stores in the municipality sell medicines.

Coahuayutla: The municipality of Coahuayutla de José Mª Izazaga, in the Costa Grande region, has a very rough, hot and semi-desert climate. In 1990 its population was reported to be 13,461. The 167 rural communities have little access to drinking water, drainage, electricity, communications or health services. Agriculture, livestock and fisheries provide employment for a very small segment of the population. The unemployed and underemployed migrate to the United States in search of better living conditions.

Xochistlahuaca: Located in the Costa Chica region, bordering the State of Oaxaca, this municipality had a population in 1990 of 16,226. Its people, mainly Amuzgo-speaking indigenous, live through subsistence farming and embroidery of women's clothes and deluxe tablecloths for a living. Unlike other indigenous groups, despite the generalised poverty that characterises the whole area, this group is known for a high degree of social integration, solidarity and low migration. In this municipality, there are 35 communities, with five health centres. Religious groups and the *Instituto Nacional Indigenista* (INI) have provided health care for the last few years.
